# Sputum colour: An indicator of *Legionella pneumophila* pneumonia

**DOI:** 10.1002/rcr2.1312

**Published:** 2024-02-28

**Authors:** Toshiyuki Sumi, Keito Suzuki, Yuta Koshino, Takumi Ikeda, Yuichi Yamada, Hirofumi Chiba

**Affiliations:** ^1^ Department of Pulmonary Medicine Hakodate Goryoukaku Hospital Hakodate Japan; ^2^ Department of Respiratory Medicine and Allergology Sapporo Medical University School of Medicine Sapporo Japan

**Keywords:** emergency department, extracorporeal membrane oxygenation, *legionella pneumophila* pneumonia, orange sputum, urine

## Abstract

The sputum colour in patients with severe pneumonia needs to be considered during diagnosis.

## Graphical abstract

We present a case of severe pneumonia with bright orange sputum. *Legionella pneumophila* pneumonia was diagnosed.

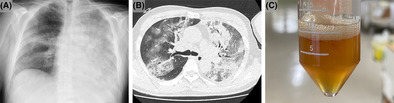

## CLINICAL IMAGE

**FIGURE 1:**
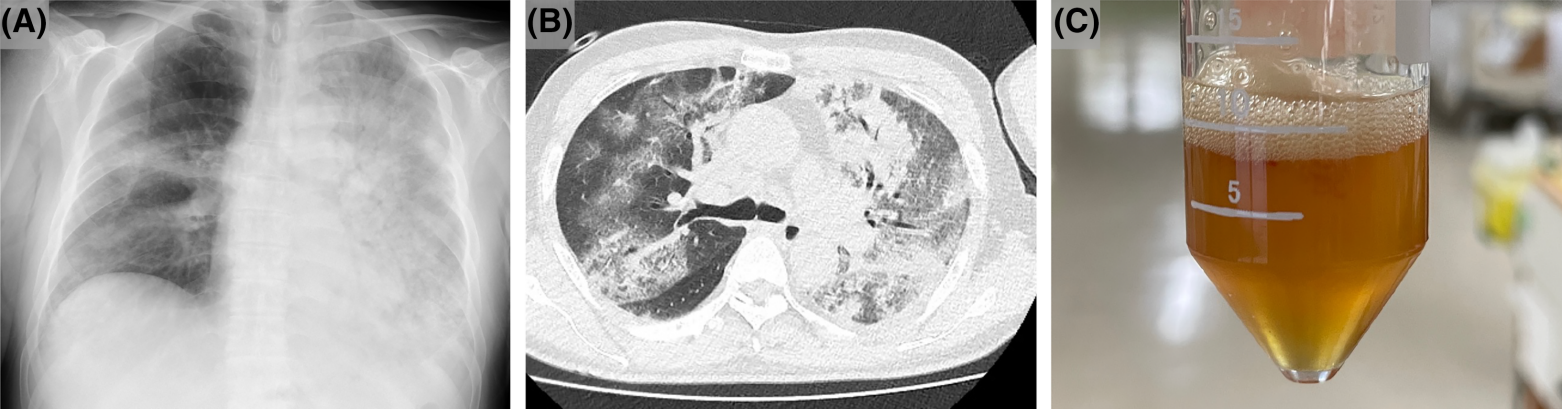
(A) Chest x‐ray shows extensive infiltrative shadows in the bilateral lungs. (B) Chest computed tomography shows extensive ground‐glass opacity and consolidation. (C) Bright orange, very watery airway secretions aspirated using a suction tube.

A 72‐year‐old Japanese man with diabetes mellitus visited the emergency department with a persistent high fever, regular heart rate (72 bpm), increased respiratory rate (28/min), and low oxygen saturation (78% in room air). Left chest auscultation revealed inspiratory coarse crackles. Blood tests revealed liver dysfunction. Chest x‐ray exhibited infiltrative shadows in the bilateral lungs (Figure 1A). Chest computed tomography showed extensive ground‐glass opacity and consolidation (Figure 1B). Intubation and ventilator management were initiated. Aspirated airway secretions were bright golden orange (Figure 1C). Sputum analysis, including BIOFIRE® Respiratory 2.1 panel, detected Legionella DNA, and urine was positive for Legionella antigen; Legionella pneumophila pneumonia was diagnosed. Such orange secretions may be produced by *L. pneumophila* by utilizing tyrosine in the epithelial lining fluid.^1^, ^2^ Despite levofloxacin treatment, respiratory failure progressed, necessitating transfer to a higher tertiary care level for extracorporeal membrane oxygenation (ECMO). ECMO, with prone therapy, steroids, and antibiotics, was initiated. Two weeks later he was tracheotomized and is undergoing rehabilitation with ventilator support. Sputum colour in pneumonia patients should be considered during diagnosis.

## AUTHOR CONTRIBUTIONS

Toshiyuki Sumi conceived the idea for the manuscript and drafted it. Keito Suzuki, Yuta Koshino, Takumi Ikeda and Yuichi Yamada contributed to the clinical management of the patient. Hirofumi Chiba revised the manuscript for intellectual content. All authors contributed to and approved the final version of the manuscript.

## CONFLICT OF INTEREST STATEMENT

None declared.

## ETHICS STATEMENT

The authors declare that appropriate written informed consent was obtained for the publication of this manuscript and accompanying images. Consent was obtained from the patient for reporting this case.

## Data Availability

The data that support the findings of this study are available from the corresponding author upon reasonable request.
